# Identification and Characterization of eccDNA in HepG2 Cells Under DOX-Induced DNA Damage

**DOI:** 10.3390/ijms262210978

**Published:** 2025-11-13

**Authors:** Jinyuan Zhang, Yuguo Li, Weijie Chen, Xingyi Du, Junnuo Zheng, Junji Chen, Xudong Huang, Chaoyang Pang, Zhiyun Guo

**Affiliations:** 1 School of Life Sciences and Engineering, Southwest Jiaotong University, Chengdu 610031, China; zjy@my.swjtu.edu.cn (J.Z.); liyuguooa@gmail.com (Y.L.); chenweijie@my.swjtu.edu.cn (W.C.); baisi0330@my.swjtu.edu.cn (X.D.); junnuo@my.swjtu.edu.cn (J.Z.); junjichen.swjtu.edu.cn@my.swjtu.edu.cn (J.C.); 2 Neurochemistry Laboratory, Department of Psychiatry, Massachusetts General Hospital and Harvard Medical School, Charlestown, MA 02129, USA; huang.xudong@mgh.harvard.edu; 3 College of Computer Science, Sichuan Normal University, Chengdu 610031, China; cypang@sicnu.edu.cn

**Keywords:** eccDNA, DNA damage, hepatocellular carcinoma, multi-omics

## Abstract

Extrachromosomal circular DNA (eccDNA) has been recognized as a key player in tumorigenesis and progression. However, eccDNA transcriptional regulatory mechanisms under DNA damage in cancer remain poorly characterized. Here, we used doxorubicin to induce DNA damage in the hepatocellular carcinoma cell line HepG2 and performed Circle-seq to profile eccDNAs before and after the damage. We observed a significant increase in the number, length, and chromosomal distribution density of eccDNAs following DNA damage. RNA-seq revealed that the expression of genes carried on eccDNA was positively correlated with eccDNA copy number under DNA damage. Further ATAC-seq profiling identified distinct chromatin characteristics at eccDNA breakpoint regions compared to other regions of eccDNA and linear genomic regions. Additionally, eccDNAs generated under DNA damage preferentially originated from linear genomic regions characterized by low GC content and hypomethylation. Finally, by integrating Hi-C and H3K27ac ChIP-seq, we uncovered that eccDNAs with mobile enhancer activity (ME-eccDNAs) display significantly enhanced chromatin interactions and H3K27ac enrichment after DNA damage. Overall, our findings systematically elucidate the DNA damage-driven mechanisms underlying eccDNA biogenesis, chromatin characteristics and transcriptional regulation in HCC HepG2 cells.

## 1. Introduction

Extrachromosomal circular DNA (eccDNA) is derived from chromosomal fragments but exists independently as circular molecules. Previous research indicates that eccDNA is detectable in nearly half of tumor cells [1]. Particularly in hepatocellular carcinoma (HCC), the existence and massive heterogeneity of eccDNAs have been observed [2], suggesting that eccDNA may play a significant role in HCC progression and prognosis [1]. Additionally, eccDNA can amplify oncogenes, providing cancer cells with survival and proliferation advantages. This amplification is further enhanced under DNA damage induced by chemotherapeutic agents, leading to drug resistance in cancer treatment [3]. Compared to other cancer types, HCC exhibits robust DNA damage repair capabilities, making it highly resistant to most conventional chemotherapies and resulting in poor treatment outcomes [4]. Previous research has indicated a close relationship between DNA damage repair pathways and eccDNA biogenesis [5,6,7]. When cells experience DNA double-strand breaks due to internal or external environmental stress, chromosomes can fragment into DNA segments of various sizes, a process known as chromothripsis. In the DNA repair process, these fragments can be rejoined into circular structures via mechanisms such as non-homologous end joining, thereby directly or indirectly facilitating eccDNA biogenesis [8]. Collectively, these findings indicate that eccDNA biogenesis and presence may be tightly linked to the cellular response and repair mechanisms to DNA damage in HCC, ultimately impacting tumorigenesis, progression, and drug resistance [9]. Therefore, in-depth research into the mechanisms of eccDNA in HCC is expected to provide new targets and strategies for HCC diagnosis and treatment.

In this study, we induced DNA damage in HepG2 cells using doxorubicin (DOX) and performed Circle-seq on cells before and after damage. We discovered significant changes in the number, length, chromosomal distribution density, GC content, and methylation levels of eccDNA in HepG2 cells following DNA damage. RNA-seq analysis revealed that not only did the copy number of eccDNA significantly increase after DNA damage, but it also showed a significant positive correlation with the expression levels of genes on the eccDNA. Functional enrichment analysis indicated that genes on eccDNA are involved in HCC-related pathways and DNA damage repair pathways. Chromatin accessibility results showed that eccDNA breakpoint regions exhibited high chromatin accessibility and nucleosome depletion both before and after DNA damage, with transcription factor enrichment at eccDNA breakpoints being more pronounced after DNA damage. Analysis of Hi-C and H3K27ac ChIP-seq data revealed that the chromatin interaction frequency of mobile enhancer-eccDNA (ME-eccDNA) regions generally increased across chromosomes after DNA damage, and the H3K27ac levels of ME-eccDNA regions were significantly higher than those of other eccDNA regions. Overall, our results highlight the critical role of DNA damage in eccDNA biogenesis and function, providing new insights into understanding HCC HepG2 cells progression mechanisms and developing eccDNA-based therapeutic strategies for HCC.

## 2. Results and Discussion

### 2.1. DOX-Induced DNA Damage Increases eccDNA Length and Biogenesis in HCC HepG2 Cells

Previous studies have shown that doxorubicin (DOX) is a common inducer of DNA damage, and the extent of DNA damage increases progressively with the duration of DOX treatment [10]. To investigate the impact of DNA damage on the biogenesis, characteristics, and functions of eccDNA in HCC, we treated HepG2 cells with 0.5 μg/mL DOX for 16 h to induce DNA damage. Circle-Seq [11] was used to detect circular DNA in both the control and DOX groups, obtaining 164.63 Mb and 171.07 Mb of clean reads from the control and DOX groups, respectively. Using Circle-Map [12], 4307 and 12,054 eccDNAs were identified in the control and DOX groups, respectively (Supplementary Table S1), indicating that DOX-induced DNA damage significantly promotes eccDNA biogenesis in HCC HepG2 cells.

**Figure 1:**
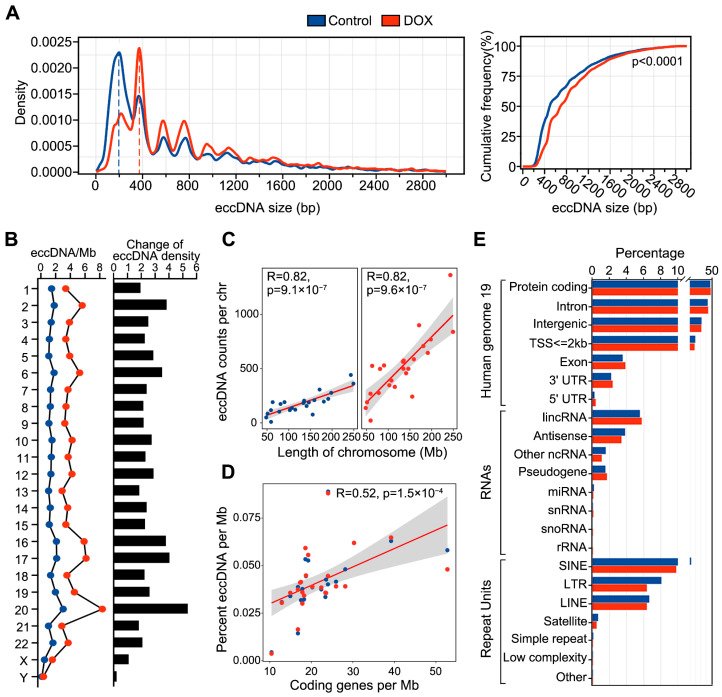
Characteristics of eccDNA in HCC HepG2 cells before and after DNA damage. (**A**), Left: The plot of eccDNA length distribution density. Right: Cumulative frequency difference in eccDNA length between control and DOX groups, using the Mann–Whitney test. (**B**), Left: Average number of eccDNAs per Mb (eccDNAs/Mb) on chromosomes. Right: Changes in eccDNA density on each chromosome before and after DNA damage. (**C**), Correlation between eccDNA abundance and chromosome length in control (left) and DOX (right) groups, using the Pearson’s test. (**D**), Correlation between eccDNA abundance and the number of coding genes per Mb, using the Pearson’s test. (**E**), Genomic annotation of eccDNA regions. Blue represents the control group, and red represents the DOX group.

Subsequently, we analyzed the characteristics of eccDNA in HCC HepG2 cells before and after DNA damage. We found that nearly all eccDNA (control, 97%; DOX, 98%) lengths ranged from 0 to 3 kb, and the DOX group showing a significant increase in eccDNA length compared to the control group (*p* < 0.0001, Mann–Whitney test) (Figure 1A). This suggests that DOX-induced DNA damage may induce new fragment fusions leading to longer eccDNAs. Additionally, both control and DOX eccDNA lengths displayed a periodic distribution with a 200 bp interval, with primary peaks around 200 bp and 400 bp, consistent with previous findings linking eccDNA biogenesis to nucleosome positioning [13,14]. Furthermore, we calculated eccDNA density on each chromosome and found that eccDNA density on all chromosomes in DOX group was higher than that in control group (Figure 1B), with chr20 showing the most significant increase. The significant increase in eccDNA density observed on chr20 is likely due to a higher degree of DNA fragmentation on this chromosome after DOX-induced DNA damage, which results in an elevated level of eccDNA formation. Pearson correlation analysis confirmed a significant positive correlation between eccDNA abundance and both chromosome length and coding gene density (Figure 1C,D).

To explore the genomic origins of eccDNA before and after DNA damage, we performed genomic annotation on eccDNAs from both groups. The results indicated that eccDNAs in both control and DOX groups primarily originated from protein-coding gene regions, intronic regions, lincRNA regions, and SINE element regions (Figure 1E). There was no notable change in the genomic distribution proportions between the two groups, suggesting that DOX-induced DNA damage does not lead to marked shifts in the genomic source of eccDNA.

### 2.2. DOX-Induced DNA Damage Significantly Affects eccDNA Copy Number and Associated Gene Expression

**Figure 2:**
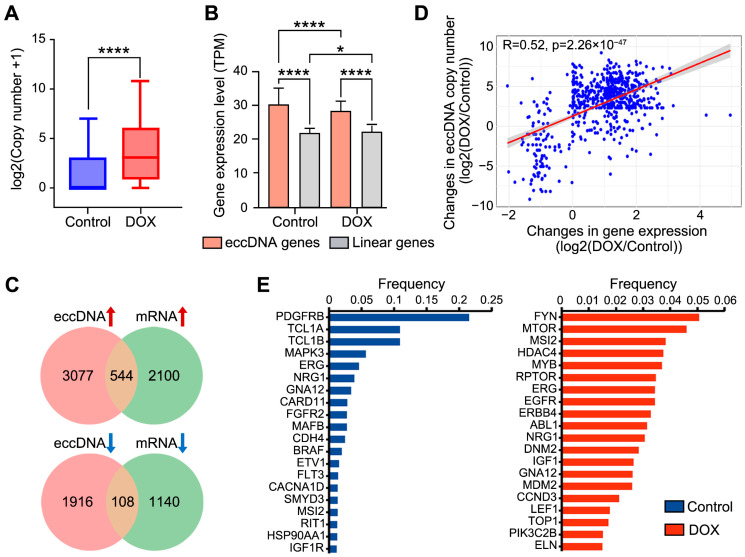
Expression analysis of eccDNA gene regions. (**A**), Comparison of eccDNA copy number before and after DNA damage. Statistical significance was assessed using paired non-parametric Wilcoxon tests. **** *p* < 0.0001. (**B**), Expression differences between eccDNA gene and linear gene regions. Statistical significance was assessed using unpaired non-parametric Kruskal–Wallis (K-W) tests. * *p* < 0.05, **** *p* < 0.0001. (**C**), Venn diagram showing the overlap of eccDNA-gene pairs with differential eccDNA copy number and differential gene expression. (**D**), Correlation between changes in eccDNA copy number and changes in gene expression, using Pearson’s test. (**E**), Amplification frequency of eccDNA oncogenes in control (left) and DOX (right) groups.

EccDNA generally exhibits higher copy numbers than linear chromosomes, thereby driving tumor progression [15]. We therefore assessed whether DNA damage in HCC HepG2 cells affects eccDNA copy number. As expected, DOX-induced DNA damage led to a significant increase in eccDNA copy number in HCC HepG2 cells (*p*-value = 1.85 × 10^−5^) (Figure 2A). The high copy number of eccDNA often results in rapid amplification of the genes located on eccDNA, thereby increasing tumor cell heterogeneity and contributing to drug resistance [16,17]. We identified genes on eccDNA, a total of 2034 genes were detected on 2168 out of 4307 eccDNAs (50.3%) in the control group, while 4659 genes were detected on 6150 out of 12,174 eccDNAs (50.5%) in the DOX group (Supplementary Table S2). Functional enrichment pathway analysis revealed that eccDNA genes were enriched in liver cancer and tumor progression-related pathways. After DOX-induced DNA damage, eccDNA genes were specifically enriched in DNA damage-related pathways, such as PI3K-Akt and mTOR pathways (Figure S1), suggesting their participation in the DNA damage response process.

To investigate whether DOX-induced DNA damage influences the expression levels of eccDNA genes, RNA-seq was performed on HepG2 cells from the control and DOX groups. The results showed that the expression levels of eccDNA genes were significantly higher than those from linear genomic regions in both groups, and the expression levels of eccDNA genes were significantly reduced after DOX-induced DNA damage (Figure 2B). EccDNA-gene pairs with synergistic changes in eccDNA copy number and gene expression, are considered to the key responders to DNA damage. Therefore, we identified 652 pairs exhibiting synergistic changes in eccDNA copy number and gene expression (544 upregulated pairs and 108 downregulated pairs) (Figure 2C) (Supplementary Table S3). Pathway enrichment analysis indicated that these 652 pairs are associated with the GTPase activity-mediated signaling pathway (Figure S2), and previous reports have suggested that GTPase activity is essential for DNA damage repair [18]. This indicates that these genes may enhance the response to DOX-induced DNA damage repair by “hitchhiking” on eccDNA. For instance, among the upregulated genes, aberrant expression of *TP53BP1* has been shown to be associated with HCC development, and defective DSB repair [19,20]. To confirm that these 652 pairs were not randomly matched, we analyzed the correlation between eccDNA copy number and gene expression, which showed a significant positive correlation (R = 0.52) (Figure 2D).

High amplification of oncogenes on eccDNA is strongly correlated with tumor development. We therefore identified eccDNA-associated oncogenes. A total of 204 eccDNA oncogenes were identified (72 in the control group and 132 in the DOX group). Pathway enrichment analysis demonstrated that eccDNA oncogenes in DOX group were extensively involved in various DNA damage pathways, whereas no such result was observed in control group (Figure S3). Moreover, amplification frequency analysis revealed substantial differences in eccDNA oncogene appearance frequency after DOX-induced DNA damage. In control group, the *PDGFRB* oncogene on eccDNA exhibited the highest amplification frequency (21.5%) (Figure 2E left), consistent with previous reports linking high *PDGFRB* amplification to enhanced tumor cell invasion and metastasis [21]. In DOX group, the *FYN* gene (5.1%) and *MTOR* oncogene (4.6%) on eccDNA showed the highest amplification frequencies (Figure 2E right). And the upregulation of *FYN* and *MTOR* has been reported to promote cancer cell survival under DNA damage induced by chemotherapy, thus exacerbating tumor progression [22,23]. In both the DOX and Control groups, *MSI2*, *ERG*, *NRG1*, and *GNA12* were commonly highly amplified oncogenes. For example, the amplification frequency of *MSI2* was significantly higher in the DOX group compared to the Control group. Previous studies have demonstrated that *MSI2*, as an oncogene, can promote hepatocellular carcinoma cell proliferation by activating the Notch1 signaling pathway [24], suggesting that its amplification may facilitate the survival of HepG2 cells under DOX-induced DNA damage.

### 2.3. EccDNA Breakpoints Are Associated with High Chromatin Accessibility and Transcription Factor Enrichment

**Figure 3:**
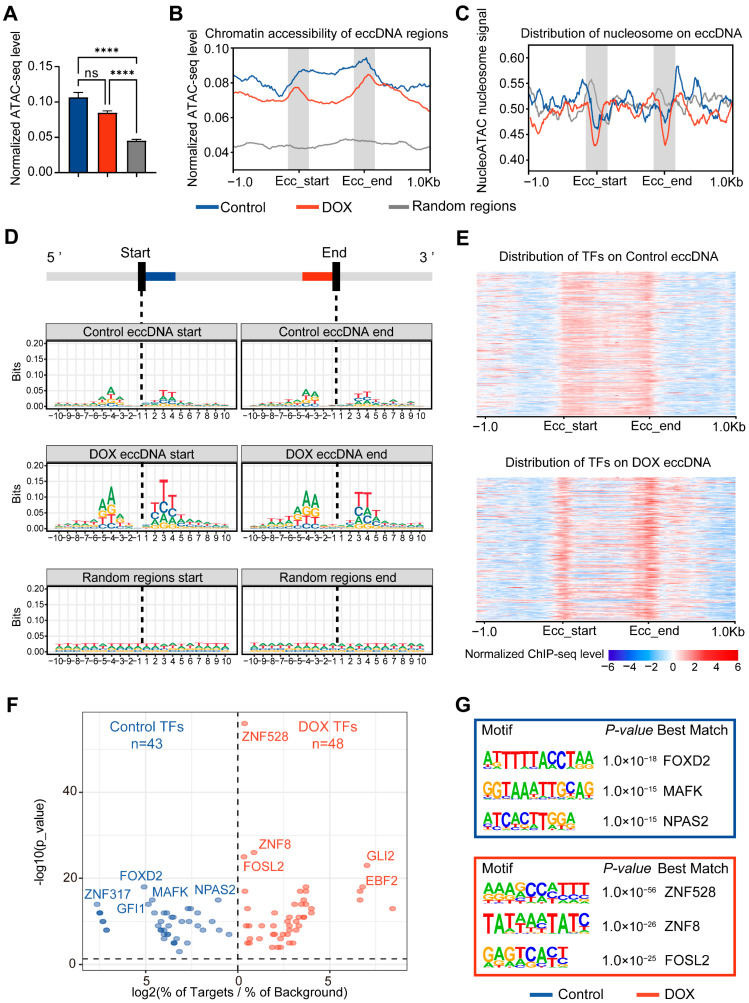
Analysis of transcription factors and accessibility at eccDNA breakpoint regions. (**A**), Chromatin accessibility of eccDNA regions before and after DNA damage. Data are presented as mean ± SEM. Statistical significance was assessed using unpaired non-parametric Kruskal–Wallis (K-W) tests. **** *p* < 0.0001. (**B**), Chromatin accessibility profile of eccDNA regions and their 1 kb upstream and downstream. “Ecc_start” and “Ecc_end” represent eccDNA breakpoints. (**C**), Nucleosome occupancy scores in eccDNA regions and their 1 kb upstream and downstream. Higher scores indicate greater likelihood of nucleosome occupancy. (**D**), Motif of 10 bp flanking eccDNA breakpoints. The height of the base letters is proportional to their conservation and frequency. (**E**), Distribution of transcription factors in eccDNA regions and their 1 kb upstream and downstream. Each row represents a transcription factor, with stronger signals indicating higher TF enrichment. “Ecc_start” and “Ecc_end” represent eccDNA breakpoints. (**F**), Volcano plot of transcription factor enrichment in eccDNA breakpoint regions. “log2(% of Targets/% of Background)” indicates (percentage of TF sequences in eccDNA breakpoint sequences)/(percentage of TF sequences in random genomic regions). (**G**), Schematic of the top 3 transcription factors related to motifs in eccDNA breakpoint regions.

Previous studies have reported that eccDNA exhibits higher chromatin accessibility compared to linear genomic regions [25]. However, it remains unclear whether DOX-induced DNA damage affects the chromatin accessibility of eccDNA regions. We therefore performed ATAC-seq on HepG2 cells before and after DNA damage. The results demonstrated that, compared with linear regions, eccDNA regions exhibit significantly higher chromatin accessibility, and DOX-induced DNA damage did not significantly alter the chromatin accessibility of eccDNA regions (Figure 3A). Notably, chromatin accessibility near eccDNA breakpoint regions was markedly higher than in other eccDNA regions (Figure 3B). Analysis of nucleosome occupancy revealed that nucleosome signals at eccDNA breakpoint flanking regions were lower than those in both linear chromosomes and other eccDNA regions, with this phenomenon becoming more pronounced after DOX-induced DNA damage (Figure 3C). These findings indicate that eccDNA biogenesis preferentially occur via breakage in highly accessible and nucleosome-free regions of linear chromosomes, this process is exacerbated by DNA damage, aligning with reports that highly accessible and nucleosome-free regions are more susceptible to damage-induced breakage [26]. To further ascertain whether these characteristics are intrinsic to the eccDNA sequence, we analyzed the nucleotide sequences within 10 bp flanking eccDNA breakpoints. The results revealed sequence conservation near the breakpoints, which became even more pronounced after DOX-induced DNA damage. Importantly, these conserved nucleotides often formed a tri-nucleotide palindromic repeat centered on the breakpoint, characteristics not observed in random genomic regions (Figure 3D), indicating unique eccDNA breakpoint characteristics.

Chromatin accessible regions are often enriched with transcription factors (TFs). We analyzed 716 TFs from the ENCODE database for HepG2 cells, finding that DOX-induced DNA damage shifted TF enrichment from a uniform distribution across eccDNA regions to a significant concentration at eccDNA breakpoint regions (Figure 3E). This suggests that these TFs bound near eccDNA breakpoints may be related to the process of DNA breakage and eccDNA biogenesis. To further explore the functions of TFs bound near eccDNA breakpoints, motif analysis was performed on 50 bp regions flanking the breakpoints (Figure 3F). The results indicated that, after DOX-induced DNA damage, various TFs related to DNA damage repair were bound to the eccDNA breakpoint regions, such as ZNF528, ZNF8 [27], and FOSL2 [28] (Figure 3G), consistent with reports linking DNA damage repair to eccDNA biogenesis [6].

### 2.4. DOX-Induced DNA Damage Leads to Decreased Genomic Stability of eccDNA Origin Linear Chromosomal Region

**Figure 4:**
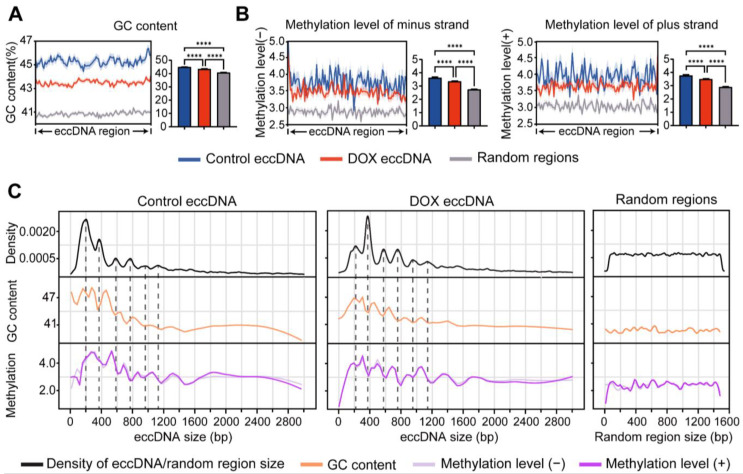
Analysis of GC content and methylation levels in eccDNA regions. (**A**,**B**), Comparison of GC content (**A**) and methylation levels (**B**) among control eccDNA, DOX eccDNA, and random genomic regions. Data are presented as mean ± SEM. Statistical significance was assessed using unpaired non-parametric Kruskal–Wallis (K-W) tests. **** *p* < 0.0001. (**C**), Periodic changes in GC content and methylation levels with eccDNA length.

DNA damage compromises the stability of linear genomes, leading to chromosomal fragmentation and concomitant eccDNA biogenesis [29]. To investigate the genomic characteristics of eccDNA origin regions under DOX-induced DNA damage, we first analyzed the GC content of these regions. The results demonstrated that, regardless of DNA damage, the GC content of eccDNA-originating regions was significantly higher than that of random linear genomic regions (Figure 4A). This indicates that eccDNA preferentially forms in GC-rich linear genomic regions. However, after DOX-induced DNA damage, the GC content of eccDNA-originating regions decreased significantly compared to control group (Figure 4A). This phenomenon may result from DNA damage compromising genomic stability, thereby allowing eccDNA biogenesis from regions with lower GC content. Previous studies have reported that GC-rich regions, particularly CpG islands, in linear chromosomes are more prone to DNA methylation [30]. To investigate whether the eccDNA-originating regions share this characteristic, we analyzed the methylation levels before and after DOX-induced DNA damage. As expected, both the positive and negative strand methylation levels of eccDNA were significantly lower after damage compared to control group, although both remained significantly higher than those of random linear genomic regions (Figure 4B). This result is consistent with the observed trend of changes in the GC content of eccDNA after DOX-induced DNA damage. The low DNA methylation state is often associated with active transcription [30]. Therefore, by integrating RNA-seq data, we observed that eccDNA regions displayed higher transcriptional activity after DOX-induced DNA damage (Figure S4), suggesting that eccDNA may modulate the transcriptional stress response after DOX-induced DNA damage. Subsequently, we further investigated the correlation between GC content and DNA methylation levels of eccDNA regions in HCC HepG2 cells. The results demonstrated that, regardless of DNA damage, a positive correlation between GC content and DNA methylation levels in eccDNA regions and random linear genomic regions (Figure S5). This finding indicates that the relationship between GC content and DNA methylation is inherent to the genomic sequence itself and is unaffected by DNA circularization or DOX-induced DNA damage.

Chromothripsis often biosynthesizes eccDNA of varying lengths. To further characterize the genomic stability of eccDNA originating from chromosomes, we categorized eccDNAs by length (0–3 kb) and examined their GC content and methylation levels. The results showed that shorter eccDNA tend to have higher GC content compared to longer eccDNA (Figure 4C), suggesting that shorter eccDNAs might be more stable. Interestingly, consistent with the observed 200 bp periodic distribution of eccDNA lengths (Figure 1A), the GC content and methylation levels of eccDNA also displayed a 200 bp periodicity. But the majority of GC and methylation peak values often opposite to eccDNA length peaks. Notably, such periodicity was not observed in random linear genomic regions (Figure 4C).

### 2.5. The Mobile Enhancer Function of eccDNA Under DOX-Induced DNA Damage

**Figure 5:**
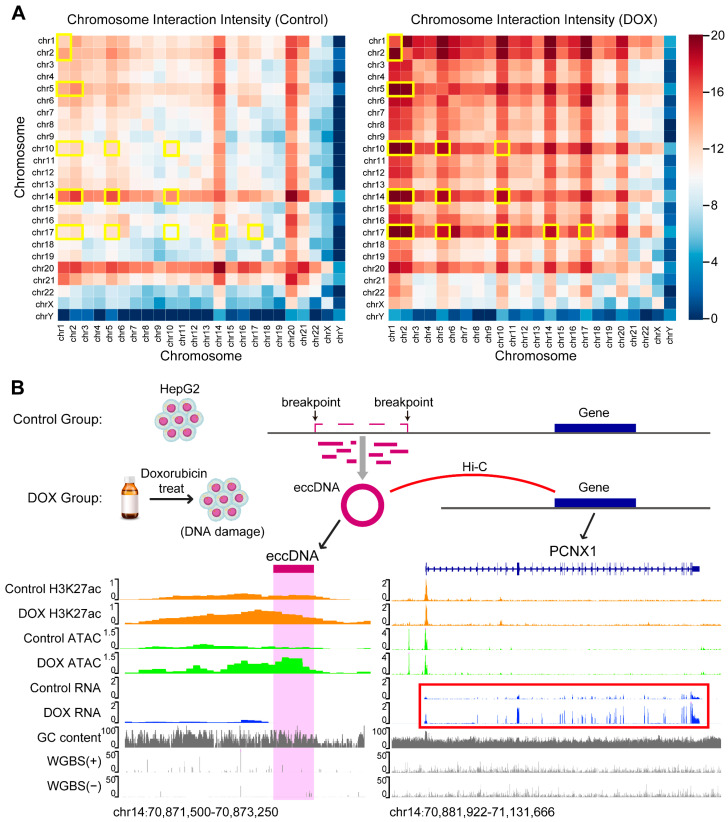
Hi-C interaction and target gene analysis of eccDNA regions. (**A**), Chromatin interaction of ME-eccDNA regions on each chromosome before and after DNA damage. The yellow boxes indicate the chromosomes in which the chromatin interactions of ME-eccDNA regions are markedly increased in the DOX group compared with the Control group. (**B**), Schematic diagram of the mechanism underlying DNA damage-induced eccDNA biogenesis. For example, the newly generated eccDNA (chr14: 70872697-70872951) after DNA damage and its regulated target gene *PCNX1* (chr14: 70907405-71115382). The H3K27ac and ATAC levels of the eccDNA (chr14: 70872697-70872951) increased, and the target gene *PCNX1* (chr14: 70907405-71115382) expression also upregulate after DNA damage (see red box). The multi-omics track diagram from top to bottom includes: H3K27ac signal, ATAC signal, RNA signal, GC content, and WGBS methylation levels.

Previous studies have shown that eccDNA can function as the mobile enhancer, interacting with linear chromosomes to regulate tumor development [31,32]. To explore how DOX-induced DNA damage influences the role of eccDNA as the mobile enhancer, we first analyzed HepG2 Hi-C data to define eccDNA regions with chromatin interactions as mobile enhancer eccDNA (ME-eccDNA). We then identified the interaction frequency of ME-eccDNA regions with other chromosomal regions. The results revealed that after DOX-induced DNA damage, the interaction frequency of ME-eccDNA regions generally increased on all chromosomes, particularly on chr1, chr2, chr5, chr10, chr14, and chr17 (Figure 5A).

Enhancer function is typically reflected by its activity, and H3K27ac level is commonly used as a marker of active enhancers [33]. To assess the impact of DOX-induced DNA damage on the activity of ME-eccDNA as the mobile enhancer, we performed H3K27ac ChIP-seq on HepG2 cells from both control and DOX groups. The results showed that ME-eccDNA regions exhibited significantly higher H3K27ac levels compared to other non-ME-eccDNA regions (Figure S6). Correlation analysis indicated that the H3K27ac levels of ME-eccDNA were strongly associated with chromatin accessibility of ME-eccDNA (Figure S7). Active enhancers typically exert their biological function by positively regulating the expression of target genes. Therefore, based on the Hi-C data, we identified 1644 and 3972 ME-eccDNA-target gene pairs in control and DOX groups, respectively (Figure 5B, Supplementary Table S4). GOBP enrichment analysis revealed that the target genes in DOX group were widely enriched in biological processes related to chromatin looping, DNA damage repair, and cell cycle regulation (Figure S8). In summary, after DOX-induced DNA damage, the interaction frequency between ME-eccDNA regions and chromosomal regions generally increased, and the H3K27ac activity of ME-eccDNA regions was significantly higher than that of other eccDNA regions. This suggests that ME-eccDNA may exert stronger mobile enhancer function after DOX-induced DNA damage.

## 3. Materials and Methods

### 3.1. Cell Culture and DOX Treatment

HepG2 cell line was obtained from the Shanghai Cell Bank of the Chinese Academy of Sciences. HepG2 cells were routinely cultured in dishes until approximately 80%. Cells were then seeded at 2.0 × 10^5^ cells per well in 6-well plates (Corning Inc., Corning, NY, USA) and cultured overnight. When cells reached approximately 70%, the medium was replaced with complete medium containing 0.5 μg/mL doxorubicin (Macklin, Shanghai, China), as described in previous research [34], and incubation continued at 37 °C in a 5% CO_2_ incubator for 16 h. Cells treated with DOX were designated as the DOX group, and untreated HepG2 cells served as the control group. Details regarding the DOX treatment and control of HepG2 cells, as well as the replicates and quality control methods for the omics sequencing, can be found in our previous publication [35].

### 3.2. Circle-seq

Samples from the control and DOX groups were subjected to Circle-seq, and these samples originated from the identical batch processed for our laboratory’s previous omics sequencing work [35]. Briefly, HepG2 cell pellets were resuspended in L1 buffer (Plasmid Mini AX; A&A Biotechnology, Gdańsk, Poland) containing proteinase K (ThermoFisher, Waltham, MA, USA) and digested overnight at 50 °C. Digested samples were subjected to alkaline treatment and column purification following the Plasmid Mini AX kit instructions. Purified DNA was digested with FastDigest MssI (ThermoFisher) at 37 °C for 16 h to remove mitochondrial eccDNA. Plasmid-Safe ATP-dependent DNase (Epicentre, Sydney, Australia) was added and incubated at 37 °C, with 30U of enzyme and corresponding ATP replenished every 24 h for a total of one week to completely remove remaining linear DNA. The treated samples were used as templates for eccDNA amplification using the RCA DNA Amplification Kit (GenSeq Inc., Dublin, Ireland), followed by purification with the MinElute Reaction Cleanup Kit (Qiagen, Hilden, Germany). Purified DNA was used for library construction with the GenSeq^®^ Rapid DNA Lib Prep Kit (GenSeq Inc.). Libraries were sequenced on the NovaSeq 6000 platform with 150 bp paired-end reads.

### 3.3. Circle-Seq Analysis

Raw reads were processed with fastp (v0.20.0) [36] to remove adapters and low-quality reads with the parameter (-q 30). Clean reads were aligned to the reference genome hg19 using BWA (v0.7.12) [37]. EccDNAs were identified using Circle-Map (v1.1.4) [12]. EccDNAs with “Split reads > 1” were retained, and the split reads for each eccDNA type were considered as the copy number, this filtering method was adapted from previous study [38]. Genomic annotations of hg19 for genes and repeat elements were annotated to eccDNA regions using BEDtools (v2.30.0) [39]. The “Simulate” tool in Circle-Map (v1.1.4) was used to generate random genomic regions, excluding sequencing gap regions from the UCSC Table Browser. Random regions ranged from 50 to 1500 bp in length and were generated in a total number of 15,000.

### 3.4. RNA-seq Analysis

The RNA-seq dataset was sourced from our previous study [35] (GSE278054). Read quality was checked with FastQC (v0.11.9). HISAT2 (v2.1.0) [40] was used to build the index for the reference genome hg19 and align sequences with default parameters. Samtools (v1.9) [41] was used for file format conversion, sorting, and indexing. FeatureCounts (v2.0.4) [42] was used for quantification with parameters (-p-countReadPairs-O). Differential gene expression analysis of eccDNA-associated genes was performed using DESeq2, with a cutoff of |log2FC| > 1 and *p*-value < 0.05. Functional enrichment (GOBP and KEGG) analyses were conducted using ClusterProfiler (v4.6.2) [43]. Pearson correlation was used to assess the relationship between gene expression changes and eccDNA copy number changes. The list of oncogenes was retrieved from OncoKB™ [44].

### 3.5. ATAC-seq Analysis

The ATAC-seq dataset was sourced from our previous study [35] (GSE278055). Raw data were processed using fastp (v0.20.0) to remove adapters. Reads were aligned to the reference genome hg19 using Bowtie2 (v2.5.1) [45], and duplicate reads were removed using Samtools (v1.9) and Picard MarkDuplicates. Peak calling was performed with MACS2 (v2.1.0) [46] using parameters (-q 0.05-f AUTO-call-summits-nomodel-shift-100-extsize 200-keep-dup all). NucleoATAC (v0.3.4) [47] was used to score nucleosome occupancy over eccDNA regions, using default parameters. The plotProfile (v3.5.1) [48] was used for visualization. The multiBigwigSummary (v3.5.1) was applied to quantify ATAC-seq signals.

### 3.6. Transcription Factor and Motif Analysis

A total of 716 transcription factor (TF) ChIP-seq datasets for HepG2 cells were downloaded from the ENCODE database. The computeMatrix (v3.5.1) was used to calculate the signal intensities for each TF in eccDNA regions and their flanking 1 kb, with parameters (-b 1000-m 1000-a 1000-bs 10). The average signal per bin was used to reflect the density of TF binding. The 50 bp regions flanking the eccDNA breakpoint were extracted, and motif enrichment analysis was performed using Homer “findMotifsGenome.pl” with parameters (-size 50-mask).

### 3.7. Analysis of GC Content and Methylation Levels

GC content data for the human genome were downloaded from the UCSC database, and methylation data for HepG2 cell line (ENCFF464CWC and ENCFF884UAE) were obtained from ENCODE. The computeMatrix (v3.5.1) was used to calculate GC and methylation levels of eccDNA with parameters (scale-regions-bs 10-beforeRegionStartLength 0-regionBodyLength 1000-afterRegionStartLength 0). Data were visualized using plotProfile (v3.5.1) with parameter (-plotType se). The multiBigwigSummary (v3.5.1) was used to quantify GC and methylation levels of eccDNA and random genomic region, and plotCorrelation (v3.5.1) was used for correlation analysis.

### 3.8. H3K27ac ChIP-seq Analysis

The H3K27ac ChIP-seq dataset was sourced from our previous study [35] (GSE278056). Raw reads were processed using fastp (v0.20.0) to remove adapters. Reads were aligned to the reference genome hg19 using Bowtie2 (v2.5.1), and peaks were called using MACS2 (v2.2.7.1) with parameters (-f BED-q 0.05-B-g hs-keep-dup all–SPMR). The multiBigwigSummary (v3.5.1) was used to quantify H3K27ac signals.

### 3.9. Hi-C Analysis and Target Gene Annotation

Hi-C data for HepG2 were downloaded from SRA (SRR1620354). Raw reads were processed with fastp (v0.20.0) to remove low-quality sequences (MAPQ < 10), and then aligned to the reference genome using Bowtie2 (v2.4.4). HiC-Pro (v3.1.0) [49] was used to generate paired contact counts. The HiC-Pro output was converted to Fit-HiC (v2.0.8) input format using “hicpro2fithic.py”, and Fit-HiC (v2.0.8) was then used to obtain intra- and inter-chromosomal interaction results. BEDtools (v2.30.0) was employed to match eccDNA regions with the Fit-HiC output to identify mobile enhancer-eccDNA (ME-eccDNA) regions and their target genomic regions. The interaction frequency of between ME-eccDNA regions and targeted chromosome regions were analyzed and chromatin interaction maps were plotted. Finally, all omics signals were visualized in IGV [50].

## Data Availability

All raw sequencing data generated in this study have been deposited in SRA, including Circle-seq (SRR33574364, SRR33574365).

## Supplementary Materials

The following supporting information can be downloaded at: https://www.mdpi.com/article/10.3390/ijms262210978/s1.
