# Supplementary material for: Identification and Characterization of eccDNA in HepG2 Cells Under DOX-Induced DNA Damage
Source: Int J Mol Sci. 2025 Nov 13;26(22):10978. doi: 10.3390/ijms262210978 (PMC12652896; doi:10.3390/ijms262210978)
Supplement: Supplementary file 1 [file ijms-26-10978-s001.zip › ijms-3930475-supplementary.pdf]

Supplementary Figures

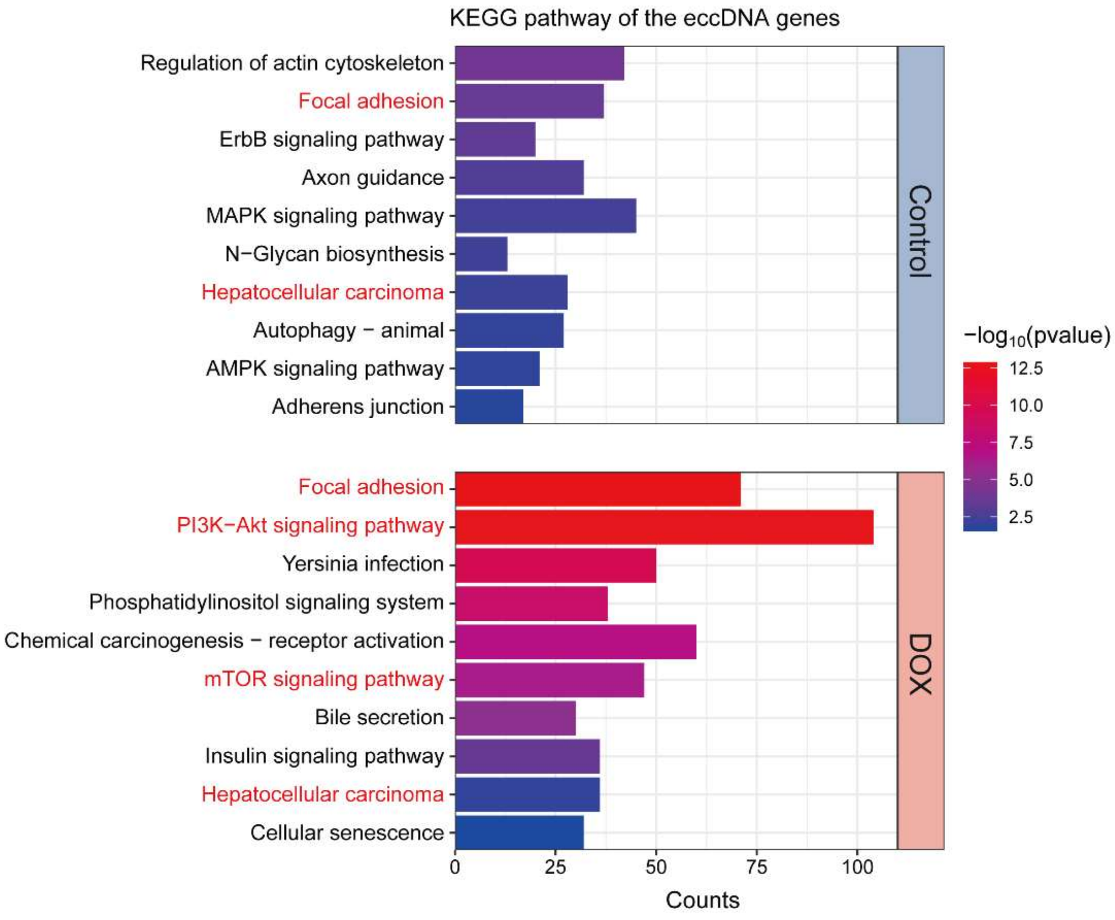

Figure S1 KEGG pathway analysis of eccDNA genes.

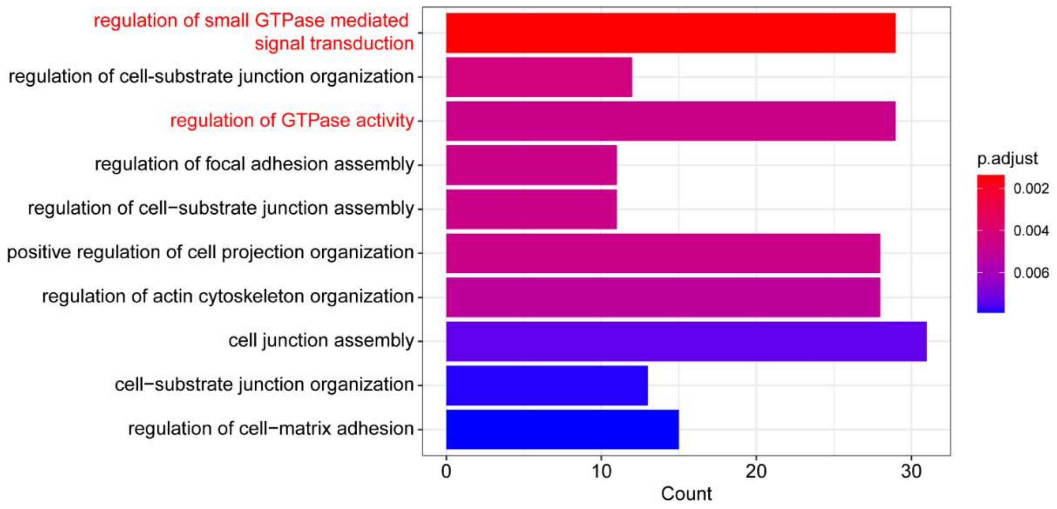

Figure S2 GOBP pathway analysis of 652 eccDNA genes with synergistic changes in eccDNA copy number and gene expression. Related to Figure 2C.

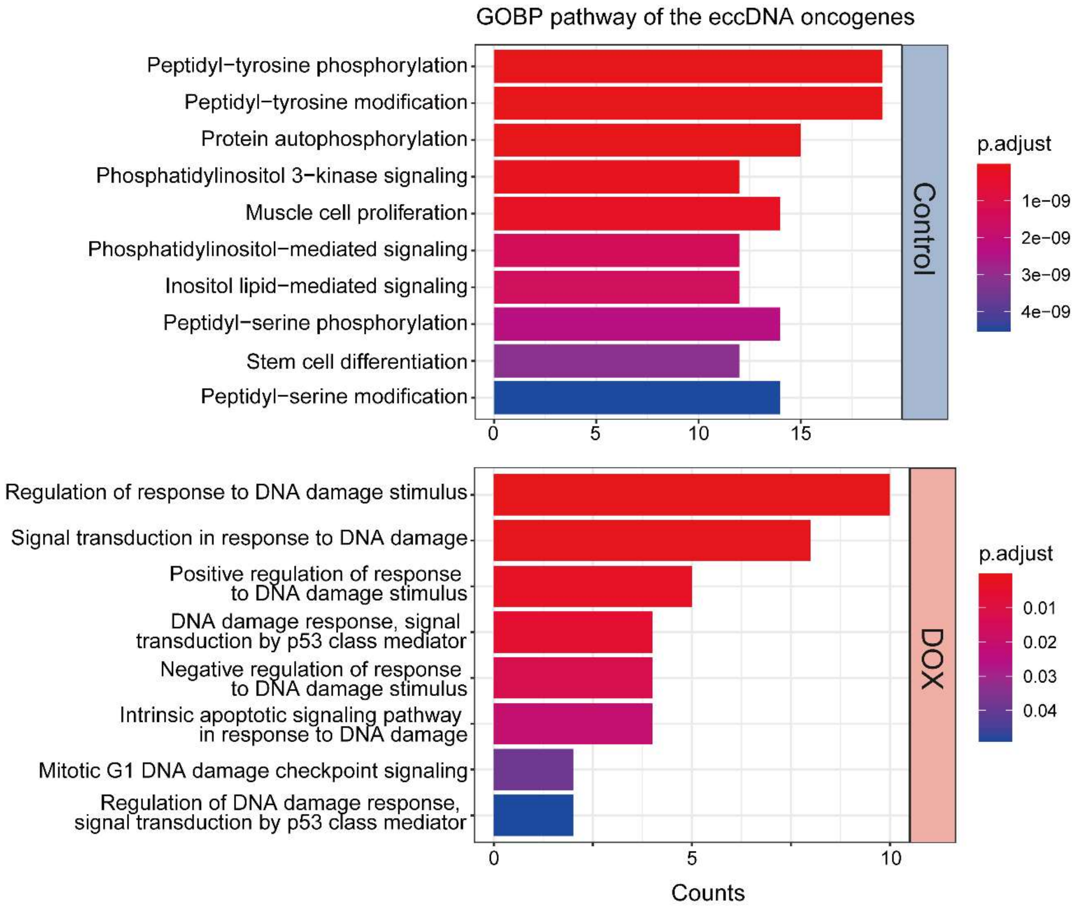

Figure S3 GOBP pathway analysis of eccDNA oncogenes. Related to Figure 2E.

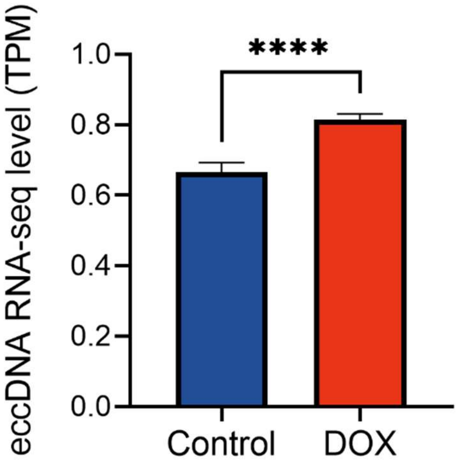

Figure S4 Changes in transcriptional expression levels (RNA-seq) in eccDNA regions during DNA damage, using t-test.

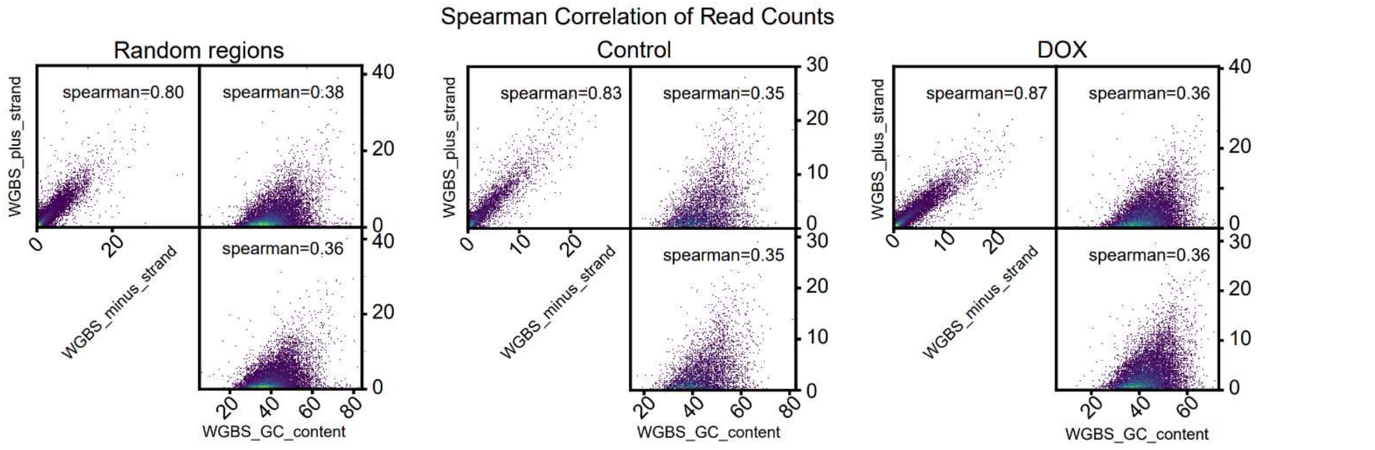

Figure S5 Correlation between eccDNA GC content and methylation levels, quantified using deepTools and analyzed for correlation using Spearman's method.

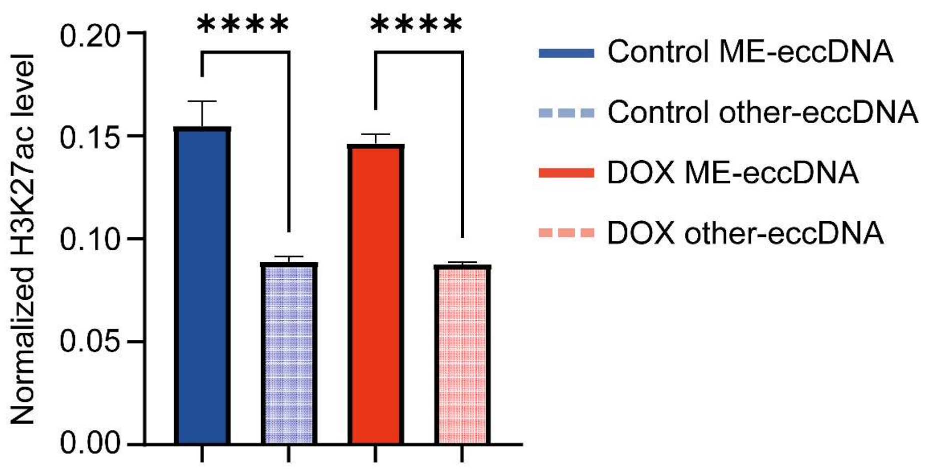

Figure S6 Comparison of H3K27ac between ME-eccDNA and other-eccDNA before and after DNA damage. Data are presented as mean ± SEM. Statistical significance was assessed using unpaired non-parametric Kruskal-Wallis (K-W) tests. $*p<0.05$, $**p<0.01$, $***p<0.001$, $****p<0.0001$.

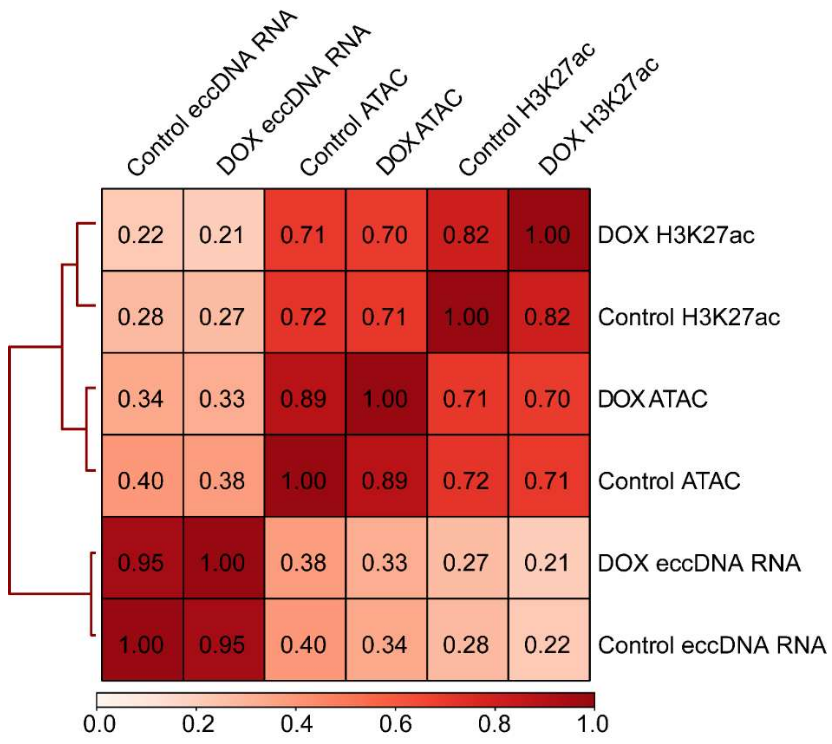

Figure S7 Correlation between multi-omics signal levels in ME-eccDNA regions.

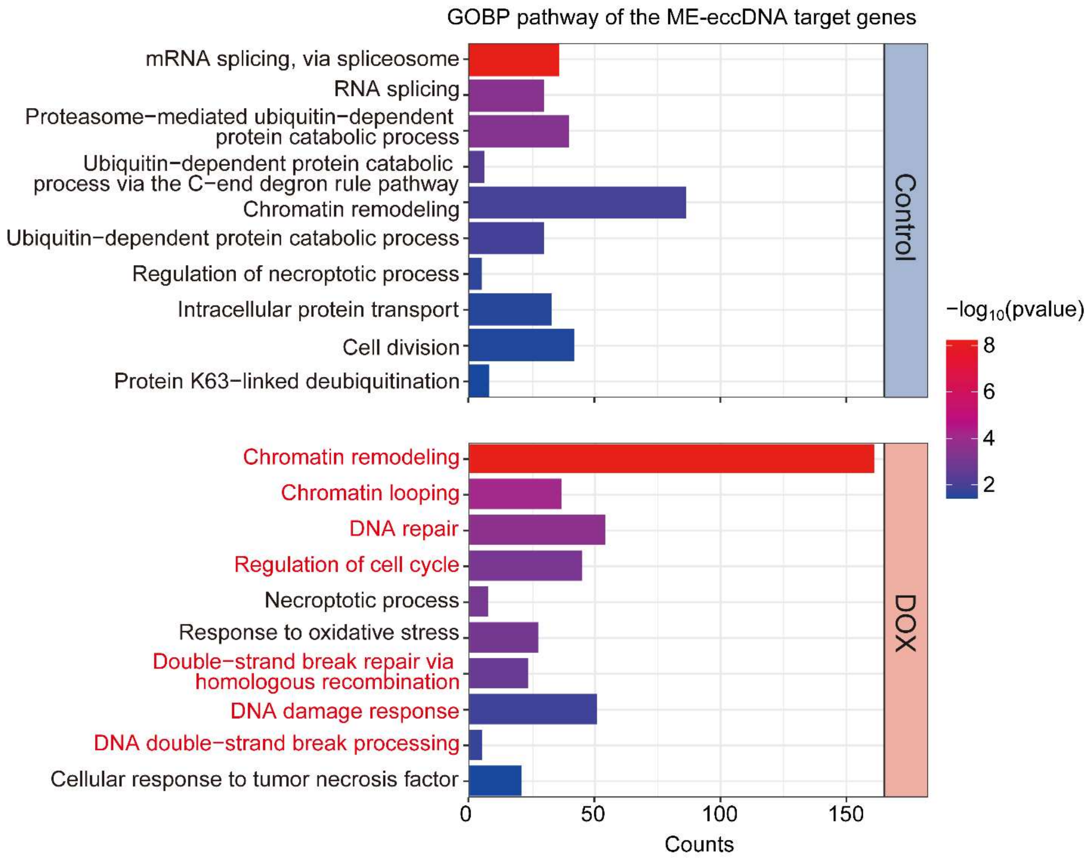

Figure S8 GOBP pathway analysis of ME-eccDNA target genes. Related to Figure 5B.
